# Bone resorption of vertebral bodies at the operative segment after prevail cervical interbody fusion: A case report

**DOI:** 10.1097/MD.0000000000035231

**Published:** 2023-09-15

**Authors:** Shufeng Shen, Yong Hu, Zhentao Chu, Weixin Dong

**Affiliations:** a Department of Spinal Surgery, Yuyao People’s Hospital, Zhejiang Province, China; b Department of Spinal Surgery, Ningbo No.6 Hospital, Zhejiang Province, China.

**Keywords:** bone resorption, case report, finite element analysis, peek prevail cervical interbody fusion

## Abstract

**Background:**

We report an interesting case of bone resorption of vertebral bodies at the operative segment after Peek Prevail cervical interbody fusion. Instability of cervical vertebrae is likely to occur due to increased stress in Peek Prevail implant body for bone resorption. The finite element analysis was used to clarify the biomechanical effects of bone resorption and stress distribution in Peek Prevail implant body.

**Methods:**

We reported the case of a 48-year-old male patient who underwent Peek Prevail cervical interbody fusion and exhibited bone resorption 1 month after the surgery in X-ray of cervical vertebra. The degree of bone resorption was aggravated 2 months after surgery. Bone resorption in 3 months was similar to that in 2 months. We established a 3D reconstruction of the surgical segment in this case using Mimics software (vision 20.0) to generate basic boss resorption model. We simulated models of bone resorption using Ansys 17.0. The stress distribution of the contact surface between the screw and bone was analyzed under 6 conditions: flexion, extension, left and right flexion, and left and right rotation.

**Results:**

The loading conditions affected the stress distribution in the implant body. When bone resorption occurred, the stress distribution of the contact surface between screw and bone focus in the tip of the screw increased sharply.

**Conclusion:**

Bone resorption of vertebral bodies in the operative segment may be a potential complication after Peek Prevail cervical interbody fusion. Great attention must be paid when bone resorption was occurred in order to avoid screw loosening before vertebral fusion.

## 1. Introduction

Anterior cervical discectomy fusion (ACDF) is usually performed to treat degenerative cervical diseases.^[1]^ ACDF includes subtotal vertebral body resection and decompression, interbody bone grafting or fusion cage placement, and titanium mesh bone grafting combined with anterior plate and screw internal fixation. However, it has been reported that this traditional technology has many potential complications, such as adjacent segment disease, loss of physiological activity, and secondary instability of adjacent segments.^[2–5]^

The Prevail interbody fusion technique was clinically used in the United States, Europe, and China in 2009, 2011, and 2014, respectively. Due to its zero notch, the implant has no contact with the soft tissue at the anterior edge of the vertebral body, reducing the incidence of dysphagia caused by anterior cervical plate implantation.^[6]^ It has certain advantages, such as shortening the operation time and reducing bleeding and operative accessory injury.^[7–9]^

Bone resorption caused by surgical implants has been commonly reported in knee arthroplasty and artificial cervical disc replacement.^[10,11]^ Bone resorption may lead to early postoperative neck pain. To the best of our knowledge, this is the first study to report bone resorption after cervical interbody fusion. In addition, a finite element analysis was used to analyze the effects of different degrees of bone resorption on the biomechanics of the internal fixation system.

## 2. Methods

### 2.1. Case presentation

**Figure 1.**
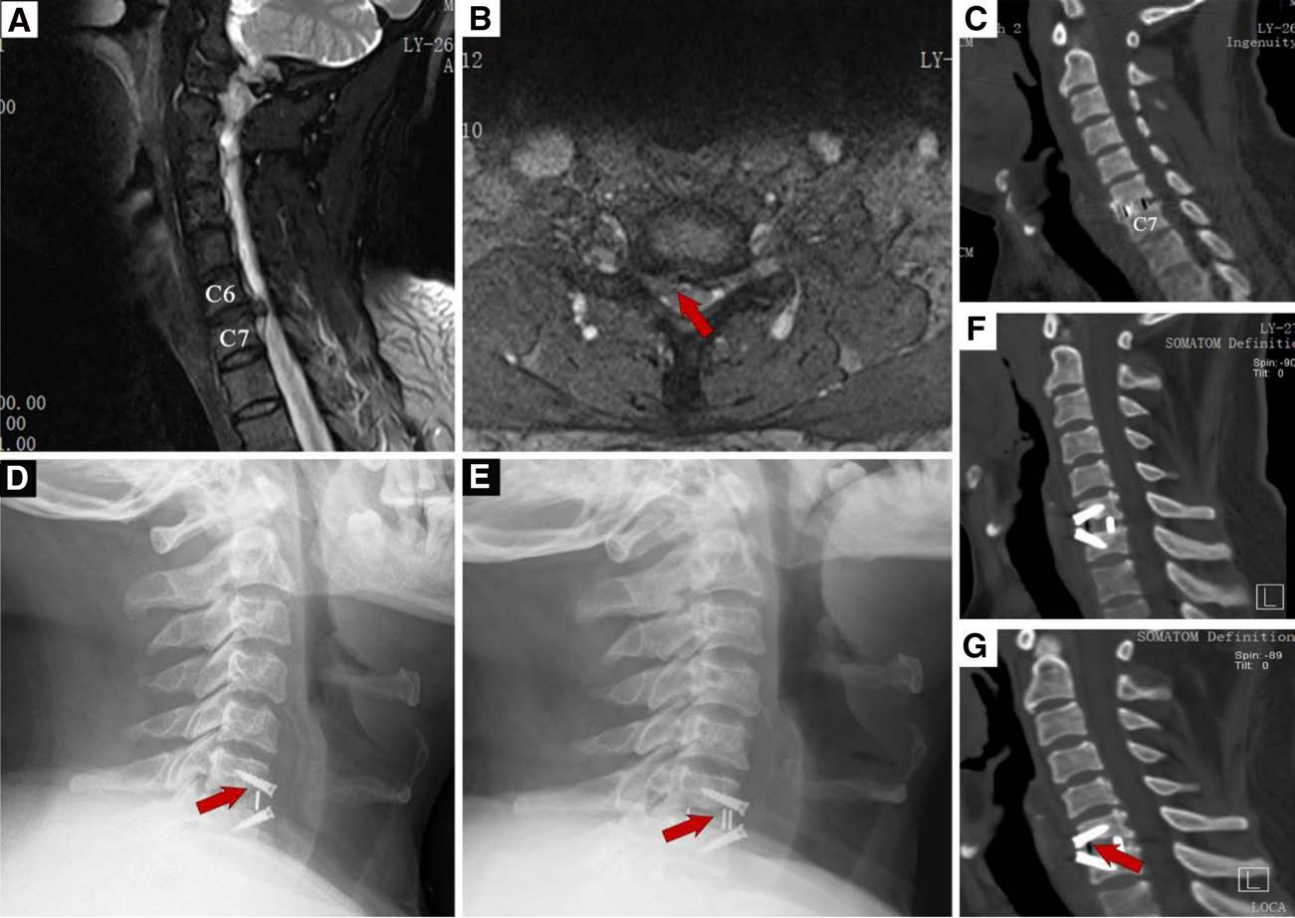
The radiology of patients was followed up before and after operation. (A–B) Preoperative magnetic resonance imaging. (C) CT imaging three days after operation. (D–E) The X-ray films of 1 month and 2 months after operation, bone resorption was indicated by red arrow. (F–G) CT three months after operation showed obvious signs of bone resorption. CT = computed tomography.

This study has been approved by the Ethics Committee of Ningbo No. 6 Hospital (Approval No.2023-16 [L]). A 48-year-old man was admitted with numbness in both lower limbs and walking instability for over 10 months. Magnetic resonance imaging showed C6/7 disc herniation, severe spinal cord compression, and degeneration of the cervical vertebrae. ACDF was performed using the Zero-Prevail device. The surgery was successful, and postoperative outcomes were satisfactory after removing the negative-pressure drainage tube. Three days after the operation, postoperative computed tomography (CT) showed no bone defects (Fig. 1A–B). Radiography revealed bone resorption at the anterior edge of C6 1 month after the operation (Fig. 1C). Two months after the operation, the radiograph showed bone resorption of the vertebral body similar to that observed 1 month after the operation (Fig. 1D–E). CT 3 months after the operation showed bone resorption at the anterior edge of the C6 vertebral body (Fig. 1F–G). The patient was instructed to wear a neck brace for 3 months, and there were no signs of screw loosening or new symptoms of nerve compression.

### 2.2. Establishment of a 3D finite element model

The preoperative CT data were imported into the Mimics software, Belgium (version 20.0) in digital imaging and communications in medicine format, preprocessing of finite element simulation was performed, the appropriate threshold was set, the surgical segment was extracted using a threshold extraction tool, and the mask was formed after region growth. A 3D reconstruction model of the surgical segment was obtained from the 3D calculations. The above model was meshed in 3-Matic software and then imported into ANSYS Workbench software for material assignment. The assignment data are listed in Table 1. The range of motion (ROM) results were compared with the biomechanical results of Moroney et al^[12]^ and Panjabi et al^[13]^

**Table 1:** Elastic modulus and Poisson’s ratio of material properties in finite element models.

Component	Elastic modulus (MPa)	Poisson ratio
Cortical bone	12,000	0.3
Cancellous bone	100	0.2
Nucleus pulposus	1.0	0.5
End plate	2000	0.2
Vertebral arch	600	0.3
Anterior longitudinal ligament	7.8	0.3
Posterior longitudinal ligament	10	0.3
Ligament flava	10	0.3
PEEK	3800	0.3
Titanium	108,000	0.3

PEEK = polyetheretherketoneketone.

**Figure 2.**
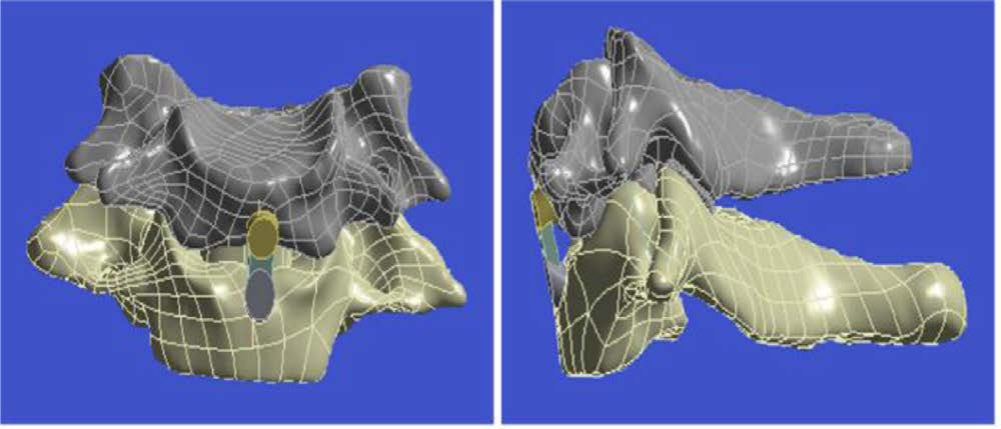
Prevail cervical interbody fusion model after meshing.

Based on the above model, the Prevail fusion model was constructed using the ANSYS Workbench software, and the final complete 3D model was obtained by addition and subtraction Boolean calculations. The 3D model of the surgical segment 3 months after the operation was calculated using the same method (Fig. 2).

The ROM and Von Mises stress of the screw of the Prevail fusion cage was assessed under the following 6 moments: flexion, extension, left/right bending, and left/right rotation. The results of these 6 moments were compared with the results of the in vitro biomechanical tests to determine whether the model was in accordance with the ROM of the normal human physiological state. To increase the comparability of the stress-Prevail fusion cage immediately and 2 months after surgery, in the 3D model, C6/7 was not regarded as a fused vertebral body 2 months after the operation.

### 2.3. Boundary and loading conditions

To make the results more comparable, the C7 vertebral body and spinous process were consistently fixed on the bottom surface in all directions, and all directions were completely constrained. The C6 endplate surface was uniformly loaded with a 75 N vertical load to simulate the cephalic weight on the cervical vertebrae.^[14]^ A 10 N.m pure torque was loaded onto C6 above the endplate surface to simulate the biomechanical characteristics of the cervical spine. The ROM and Von Mises stress of the screw of the Prevail fusion cage under flexion, extension, left/right bending, and left/right rotation were obtained in the normal and bone resorption models.

## 3. Results

### 3.1. The validation of the finite element model

**Figure 3.**
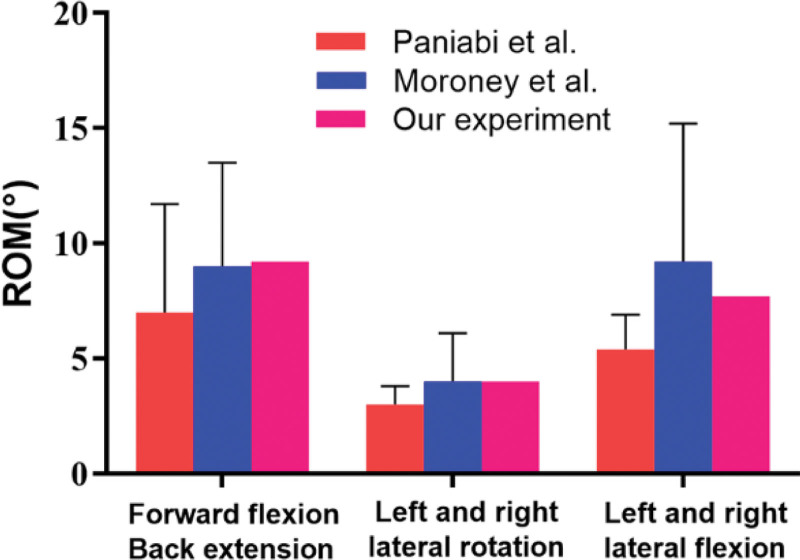
Validation of the model and ROM showed the degree of intervertebral motion. ROM = range of motion.

A normal finite element model of C6/C7 was established. For model validation, a comparison between the biomechanical results obtained by Moroney et al^[12]^ and Panjabi et al^[13]^ (Fig. 3) was conducted, from which it could be concluded that the ROM of the normal finite element model of C6/C7 in this study was reasonable for biomechanical measurements in vitro.

### 3.2. Changes in ROM after bone resorption occurred 2 months after the operation

**Figure 4.**
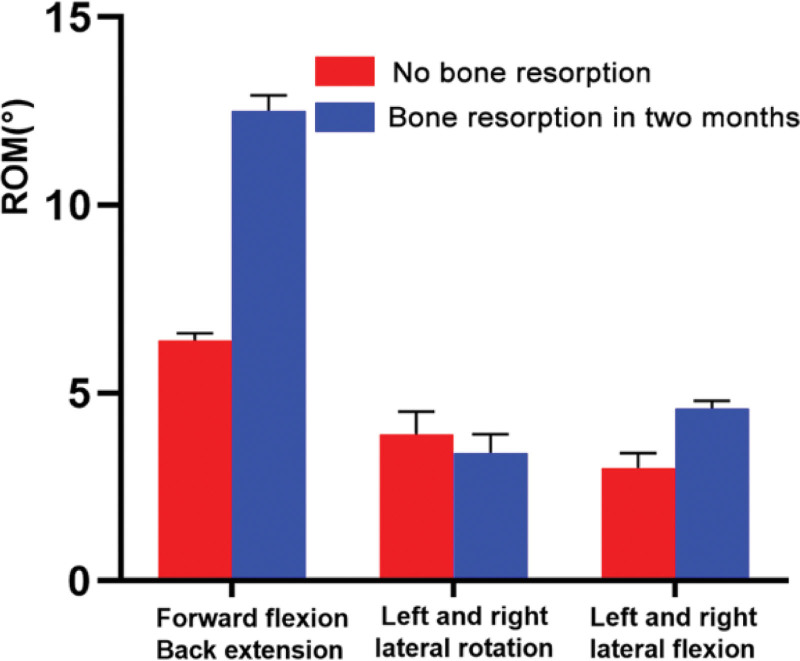
Comparison of ROM on the model between no bone resorption and bone resorption in two months. ROM = range of motion.

Under 75 N axial pressure and 1 N.m pure coupled moment load, the ROM of the bone resorption model was significantly higher than that without bone resorption under flexion, extension, lateral flexion, and rotation, especially in the flexion and extension positions (Fig. 4). Compared with the model immediately after the operation, the overall ROM of bone resorption model increased by approximately 6º and 2º in flexion and extension and lateral flexion, respectively.

### 3.3. Equivalent stress in the prevail screws

**Figure 5.**
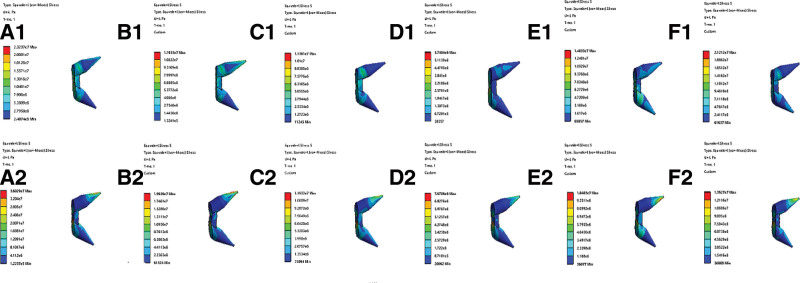
Comparable nephogram of equivalent stress in the prevail screws between no bone resorption (the pictures above) and bone resorption two months after operation (the pictures below). (A) Forward flexion, (B) back extension, (C) lateral flexion of plasty, (D) lateral flexion opposite plasty, (E) lateral rotation of plasty, and (F) lateral rotation opposite plasty.

The von Mises stress around the screw increases when bone resorption occurs (Fig. 5). In all working tasks, the maximum stress of the screw appeared in the tail section of the screw (where the diameter was small). Stress changes were evident during the flexion and extension tasks. In the forward flexion state, the stress at the tail end of the screw increased by 64.7%, from 1.19 Mpa to 1.96 Mpa. In the back extension state, the stress at the tail end of the screw increased from 2.32*10^4^ Mpa to 3.61 Mpa, an increase of 55.2%.

## 4. Discussion

Compared to the traditional titanium plate fusion cage decompression and internal fixation system, satisfactory clinical results and neurological function recovery were obtained with the Prevail cervical interbody fusion system. At the same time, it has the advantages of a small incision, short operation time, less intraoperative bleeding, and convenient operation, and it can effectively reduce the incidence of postoperative dysphagia and the loss of intervertebral height in fusion segments.^[6,15,16]^ Despite the large number of reports on its advantages, there are few reports on complications related to preventing cervical interbody fusion systems. Shi et al^[17]^ reported a case of allergic symptoms, such as throat pruritus, eyeball congestion, and skin rash, 1 month after ACDF with a Zero-P device. In this study, we report a case of vertebral bone resorption after the implantation of a Prevail fusion cage and study the changes in Von Mises stress and vertebral ROM in the case of bone resorption using the finite element method. To the best of our knowledge, this is the first report of bone resorption around a screw in a Prevail cervical interbody fusion system. Currently, bone resorption is more common in large joint replacements such as hip and knee joint arthroplasty. Several studies have reported cases of bone resorption after cervical total disc replacement. The reported incidence of bone resorption is 4.3% to 60.4%.^[18–20]^ In 2017, our hospital began using the Prevail technology, and approximately 300 cases of single-segment or double-segment cervical ACDF surgery have been performed. Each patient was followed up for at least 3 months. Each patient underwent a cervical CT examination 3 months postoperatively. Except for one case reported in this study, no bone resorption around the screw was found in the other patients.

In this case, the radiographic features of bone resorption were quite different from those previously reported after total cervical disc replacement. In this case, the bone resorption around the screw presented a conical shape from the outside to the inside, with the screw at the center, resulting in defects in the bone cortex and cancellous bone, making it more prone to vertebral instability before vertebral fusion. In the latter, bone resorption after intervertebral disc replacement mostly occurs at the anterior edge of the inferior vertebral body and anterior osteophyte loss or bone loss of a small portion of the anterior margin of the vertebral bodies of the operative segment.^[18]^ The biggest difference from this case was that the bone cortex of the cervical vertebra was intact. Therefore, we speculate that the mechanism of bone resorption, in this case, may be different from that of artificial disc replacement, and further studies are required.

The finite element analysis results showed that when bone resorption occurred around the screw, the loss of bone support around the screw increased the overall stress on the vertebral body and screw, which may lead to screw loosening or even fracture, thus affecting the stability of the corresponding segments. In this case, during the follow-up 1 and 2 months after the operation, possible bone resorption was observed during the X-ray examination in the author’s outpatient clinic, and the patient was firmly instructed to wear a neck brace. Moreover, a doctor is seen when there are neurological symptoms, such as cervical pain and numbness of both upper limbs. Fortunately, a bony fusion of C6/C7 was observed on CT 3 months postoperatively. The patients were instructed to wear a neck brace for 1 month. There was no neck pain or nerve compression recurrence during the follow-up period up to 1 year after the operation. Therefore, the author concludes that for patients with Prevail cervical fusion, it is necessary to regularly follow-up for osseous fusion after surgery. For patients with potential bone resorption, the neck brace should be worn strictly, and the wearing time should be extended appropriately.

## 5. Limitations

There are some limitations in this study. First, when establishing the finite element model, the lower endplate of C7 was regarded as fixed, which is different from the actual activity of the cervical vertebra; however, this difference can be ignored by analyzing the model’s effectiveness. In addition, more objective and rigorous diagnostic and classification criteria are required for bone resorption to provide a reference for clinical applications.

## 6. Conclusion

Bone resorption of the vertebral bodies in the operative segment may be a potential complication after Peek Prevail cervical interbody fusion. Compared with the status without bone resorption, the ROM with bone resorption was significantly increased, and the force around the screw was significantly increased. We suggest that when bone resorption occurs, the doctor should firmly instruct the patient to strictly wear a neck brace and visit the clinic for timely reexamination.

## Author contribution

**Conceptualization:** Shufeng Shen.

**Data curation:** Shufeng Shen.

**Formal analysis:** Shufeng Shen, Yong Hu.

**Investigation:** Shufeng Shen, Weixin Dong.

**Methodology:** Shufeng Shen.

**Project administration:** Shufeng Shen, Weixin Dong.

**Resources:** Shufeng Shen.

**Supervision:** Yong Hu.

**Software:** Zhentao Chu.

**Validation:** Zhentao Chu.

**Visualization:** Yong Hu.

**Writing – original draft:** Shufeng Shen.

**Writing – review & editing:** Yong Hu, Zhentao Chu.
